# Early mobilization in the time of COVID-19

**DOI:** 10.5935/0103-507X.20200086

**Published:** 2020

**Authors:** Kelly Cattelan Bonorino, Katerine Cristhine Cani

**Affiliations:** 1 Adult Intensive Care Unit, Hospital Universitário Polydoro de Ernani de São Thiago, Universidade Federal de Santa Catarina - Florianópolis (SC), Brazil.; 2 Department of Physical Therapy, Centro de Ciências da Saúde e do Esporte, Universidade do Estado de Santa Catarina - Florianópolis (SC), Brazil.

## INTRODUCTION

It is essential to consider the deleterious secondary effects of coronavirus 2019 (COVID-19) disease and its consequences, especially in patients who develop the most severe forms. The survival of acute critical illness in the intensive care unit (ICU) may not reflect the patient’s quality of life after hospitalization.^(1)^

A study with survivors of acute respiratory distress syndrome (ARDS) found that 24 months after the disease, these patients had significantly lower exercise capacity and health status than healthy individuals. In addition, 29% of the survivors had not returned to work.^(2)^

The interactions among the critical illness-related complications, comorbidities, life-support treatments, organizational aspects of intensive care and adaptation during the post-ICU period may contribute to the development of post-intensive care syndrome.^(1,3)^ This syndrome is characterized by physical, functional, cognitive and psychiatric changes and by the development of posttraumatic stress disorder, which can lead to reduced quality of life.^(4,5)^

In this context, it is suggested that the repercussions of COVID-19 resulting from treatment in the ICU include the development of ICU-acquired weakness (ICUAW) and that its consequences extend beyond the hospitalization period.

### Risk factors for intensive care unit-acquired weakness in COVID-19

Many patients infected with COVID-19 require ICU admission due to severe acute respiratory failure and the development of ARDS, which is considered a risk factor for ICUAW. In addition, a systemic inflammatory process occurs via the release of proinflammatory cytokines, which contribute to the mechanism of muscle mass loss.^(6)^ In association with these factors, the duration of mechanical ventilation - another factor associated with ICUAW - for these patients is high (mean of 11.7 days)^(7)^, as are the lengths of stay in the ICU and hospital.^(8)^ Approximately 75-80% of patients hospitalized with COVID-19 have prolonged hospital stays of approximately 21 days.^(9)^

**Figure 1:**
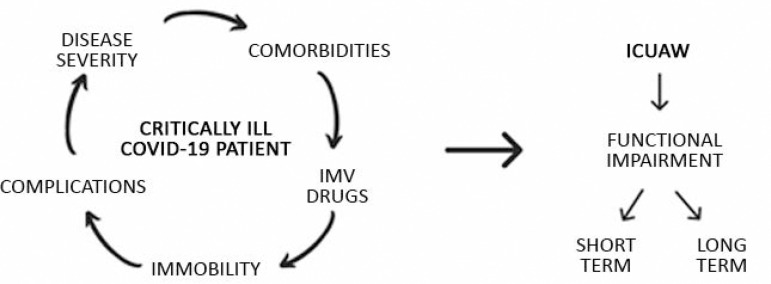
Vicious cycle that contributes to the development of intensive care unit-acquired weakness and functional disorders in the short and long term in critically ill patients. IMV - invasive mechanical ventilation; ICUAW - intensive care unit-acquired weakness.

Patients with COVID-19 who are admitted to the ICU may have (multiple) organ failure, including ARDS, acute kidney injury, cardiac injury and liver dysfunction.^(10)^ Evidence has shown that organ dysfunction is strongly associated with muscle dysfunction.^(11)^ In addition, some of these patients have associated comorbidities, such as advanced age, renal dysfunction, hypertension, diabetes, heart disease, which may contribute to the incidence of ICUAW.^(8,9)^ All these factors contribute to immobility, which, in turn, has deleterious effects on the cardiorespiratory, central nervous, musculoskeletal systems and metabolism.^(12)^Thus, critically ill patients with COVID-19 may face a vicious cycle in which disease severity, the presence of comorbidities, prolonged invasive ventilatory support and the use of sedatives and neuromuscular blockers may contribute to the development of ICUAW and functional disorders in the short and long term (Figure 1).

Thus, these risk factors should be used as a means of evaluation and screening of these patients to promote early rehabilitation through early mobilization (EM) protocols in order to avoid and/or minimize complications and functional decline.

### Early mobilization in patients with COVID-19

Considering the clinical conditions caused by prolonged immobility and musculoskeletal deterioration, the implementation of systematized EM protocols is of fundamental relevance for patients with COVID-19, given the growing evidence of its benefit.^(13,14)^EM helps to reduce the deleterious effects of the disease, especially on muscle and cardiopulmonary function, mobility and functionality. It is a safe and feasible practice that leads to improved muscle strength and functional recovery with improved quality of life.^(14,15)^ EM also leads to better clinical outcomes, such as reduced mechanical ventilation durations and lengths of ICU and hospital stay.^(14,15)^Thus, it helps to reduce hospital costs and may also prevent readmission to the ICU/hospital.^(16)^

Currently, we are experiencing an overload of the health system, and ICUs are experiencing high occupancy rates or are at maximum capacity in Brazilian states; this leads to work overload for health professionals, given that ICUs are often not large enough to meet the demand.^(17)^ It is known that in such settings, the priority may be the provision of advanced ventilatory care. However, rehabilitation should be incorporated into pandemic response plans at the onset of hospitalization and not only after patients experience consequences. In such cases, the multidisciplinary team plays a crucial role in the functional recovery and reintegration of these individuals into society.^(18)^

It is suggested that early rehabilitation interventions in patients with COVID-19, especially those who develop with severe muscle dysfunction, fatigue and dyspnea,^(13)^ be initiated during hospitalization and continue in specialized rehabilitation programs after discharge in order to improve their functionality and quality of life and prevent rehospitalization.

### Strategies for implementing early mobilization in COVID-19

Physical therapists have a role in providing interventions for mobilization, exercise and rehabilitation, especially for patients at risk of developing functional decline. In this context, strategies and recommendations were developed for workforce planning and preparation.^(19-21)^

Planning for the number of professionals needed to implement comprehensive patient care in the ICU and on the wards is recommended. The inclusion of professionals who have experience caring for critically ill patients in a hospital environment should be prioritized, i.e., physical therapists must have specialized knowledge, skills and decision-making capacity.^(19-21)^

Physical therapists with previous ICU experience should be identified and the return of these professionals to the ICU should be facilitated. It is also recommended that professionals who have no experience in the ICU be identified and used to support the care of COVID-19 patients in other areas of the hospital.^(19-21)^

Another important point is the implementation of training and learning resources, such as the development and management of critical care skills and training on the use of personal protective equipment.^(19-21)^

Lastly, to minimize the impact of COVID-19 on patients undergoing home treatment postdischarge, televisits, telehealth consultations and telemonitoring services may be important treatment tools.^(22)^ Thus, we suggest that future studies should develop similar approaches in Brazil to better elucidate the effects of COVID-19 on the functionality of affected individuals.

## References

[1] Robinson CC, Rosa RG, Kochhann R, Schneider D, Sganzerla D, Dietrich C (2018). Qualidade de vida pós-unidades de terapia intensiva: protocolo de estudo de coorte multicêntrico para avaliação de desfechos em longo prazo em sobreviventes de internação em unidades de terapia intensiva brasileiras. Rev Bras Ter Intensiva.

[2] Ngai JC, Ko FW, Ng SS, To KW, Tong M, Hui DS (2010). The long-term impact of severe acute respiratory syndrome on pulmonary function, exercise capacity and health status. Respirology.

[3] Carson SS, Bach PB, Brzozowski L, Leff A (1999). Outcomes after long-term acute care: An analysis of 133 mechanically ventilated patients. Am J Respir Crit Care Med.

[4] Needham DM, Wozniak AW, Hough CL, Morris PE, Dinglas VD, Jackson JC, Mendez-Tellez PA, Shanholtz C, Ely EW, Colantuoni E, Hopkins RO, National Institutes of Health NHLBI ARDS Network (2014). Risk factors for physical impairment after acute lung injury in a national, multicenter study. Am J Respir Crit Care Med.

[5] Myhren H, Ekeberg O, Toien K, Karlsson S, Stokland O (2010). Posttraumatic stress, anxiety and depression symptoms in patients during the first year post intensive care unit discharge. Crit Care.

[6] Jose RJ, Manuel A (2020). COVID-19 cytokine storm: the interplay between inflammation and coagulation. Lancet Respir Med.

[7] UTIs Brasileiras Registro Nacional de Terapia Intensiva. Brazilian ICUs project.

[8] Yang X, Yu Y, Xu J, Shu H, Xia J, Liu H (2020). Clinical course and outcomes of critically ill patients with SARS-CoV-2 pneumonia in Wuhan, China: a single-centered, retrospective, observational study. Lancet Respir Med.

[9] Wang L, He W, Yu X, Hu D, Bao M, Liu H (2020). Coronavirus disease 2019 in elderly patients: characteristics and prognostic factors based on 4-week follow-up. J Infect.

[10] Gupta A, Madhavan MV, Sehgal K, Nair N, Mahajan S, Sehrawat TS (2020). Extrapulmonary manifestations of COVID-19. Nat Med.

[11] Puthucheary ZA, Rawal J, McPhail M, Connolly B, Ratnayake G, Chan P (2013). Acute skeletal muscle wasting in critical illness. JAMA.

[12] Nava S, Piaggi G, De Mattia E, Carlucci A (2002). Muscle retraining in the ICU patients. Minerva Anestesiol.

[13] Aquim EE, Bernardo WM, Buzzini RF, Azeredo NS, Cunha LS, Damasceno MC (2019). Brazilian Guidelines for Early Mobilization in Intensive Care Unit. Rev Bras Ter Intensiva.

[14] Kayambu G, Boots R, Paratz J (2013). Physical therapy for the critically ill in the ICU: a systematic review and meta-analysis. Crit Care Med.

[15] Connolly B, O'Neill B, Salisbury L, Blackwood B, Enhanced Recovery After Critical Illness Programme Group (2016). Physical rehabilitation interventions for adult patients during critical illness: an overview of systematic reviews. Thorax.

[16] Hunter A, Johnson L, Coustasse A (2014). Reduction of intensive care unit length of stay: the case of early mobilization. Health Care Manag (Frederick).

[17] Associação Brasileira de Fisioterapia Cardiorrespiratória e Fisioterapia em Terapia Intensiva (2020). COVID-19 Mobilização precoce na insuficiência respiratória aguda: IRpA. Comunicação oficial..

[18] Simpson R, Robinson L (2020). Rehabilitation after critical illness in people with COVID-19 infection. Am J Phys Med Rehabil.

[19] Thomas P, Baldwin C, Bissett B, Boden I, Gosselink R, Granger CL (2020). Physiotherapy management for COVID-19 in the acute hospital setting: clinical practice recommendations. J Physiother.

[20] The Chartered Society of Physiotherapy (2020). Rehabilitation and Covid-19. CSP policy statement.

[21] Spruit MA, Holland AE, Singh SJ, Troosters T (2020). Report of an ad-hoc international task force to develop an expert-based opinion on early and short-term rehabilitative interventions (after the acute hospital setting) in COVID-19 survivors.

[22] Conselho Federal de Fisioterapia e Terapia Ocupacional Resolução Nº 516, de 20 de março de 2020. Teleconsulta, Telemonitoramento e Teleconsultoria. Dispõe sobre a suspensão temporária do Artigo 15, inciso II e Artigo 39 da Resolução COFFITO nº 424/2013 e Artigo 15, inciso II e Artigo 39 da Resolução COFFITO nº 425/2013 e estabelece outras providências durante o enfrentamento da crise provocada pela Pandemia do COVID-19.

